# Commentary: Effect of Levothyroxine on Miscarriage among Women with Normal Thyroid Function and Thyroid Autoimmunity Undergoing *In Vitro* Fertilization and Embryo Transfer: A Randomized Clinical Trial

**DOI:** 10.3389/fendo.2018.00073

**Published:** 2018-03-05

**Authors:** Spyridoula Maraka, Derek T. O’Keeffe, Naykky Singh Ospina

**Affiliations:** ^1^Division of Endocrinology, Medicine, University of Arkansas for Medical Sciences, Little Rock, AR, United States; ^2^Medicine, Central Arkansas Veterans Healthcare System (CAVHS), Little Rock, AR, United States; ^3^KER-Endo, Medicine, Mayo Clinic, Rochester, MN, United States; ^4^Division of Endocrinology, Medicine, National University of Ireland Galway, Galway, Ireland; ^5^Division of Endocrinology, Medicine, University of Florida, Gainesville, FL, United States

**Keywords:** thyroid hormone replacement, levothyroxine, thyroid autoimmunity, miscarriage, pregnancy, live-birth rate, *in vitro* fertilization

In a recent issue of JAMA, we read with interest the results of The Pregnancy Outcomes Study in Euthyroid Women with Thyroid Autoimmunity after Levothyroxine (POSTAL) study (1). Thyroid autoimmunity, as reflected by anti-thyroperoxidase antibodies (TPOAb) or anti-thyroglobulin antibodies (TgAb) positivity, is common in women of reproductive age, with an estimated prevalence of up to 14% (2). Thyroid autoimmunity is associated with infertility and a higher risk of miscarriage and premature delivery (3–5). However, this association does not prove causality and so far, there is no clear biologic plausibility. One of the mechanistic hypotheses is that the thyroid autoimmunity leads to mild thyroid hypofunction that subsequently creates a suboptimal environment during the early phase of implantation (6), which may increase the risk for infertility or miscarriage. Taking this into consideration, an important research question is whether levothyroxine (LT4) treatment could mitigate these adverse events. However, to assess if LT4 treatment for patients with thyroid autoimmunity affects the miscarriage rate and to identify the optimal potential treatment window, studies including women at early pregnancy are pivotal.

Women undergoing fertility treatment is an excellent population in which to study the effects of LT4 treatment on early reproduction outcomes, such as miscarriage. A retrospective cohort study (*n* = 93), where LT4, acetyl-salicylic acid, and prednisolone were given in a non-randomized design, showed a similar miscarriage rate between euthyroid women with thyroid autoimmunity who received LT4 (27.3%) and those who did not (25%) (7). Similarly, results from a randomized clinical trial (*n* = 72) showed that LT4 treatment for thyroid Ab-positive women with normal thyroid function undergoing assisted reproductive technology (ART) did not improve outcomes (miscarriage rate 33% treated vs 52% untreated) (8). Both studies were limited by small sample size and design issues. The American Thyroid Association (ATA) found this evidence insufficient to determine whether LT4 therapy improves the success of pregnancy following ART in TPOAb-positive euthyroid women (9). The question remained whether the guidelines would have been different if these studies were adequately powered and designed differently. Therefore, the 2017 guideline includes that administration of 25–50 μg LT4 to TPOAb-positive euthyroid women undergoing ART may be considered given its potential benefits compared to its minimal risk (9).

The POSTAL study sought to assess whether LT4 treatment initiated before *in vitro* fertilization and embryo transfer (IVF-ET) could decrease the miscarriage and improve the live-birth rates among euthyroid women with positive TPOAb (1). Participants were randomized to receive either LT4 or the blank control. Women randomized to the intervention group began LT4 treatment 2–4 weeks before the controlled ovarian hyperstimulation allowing adequate time for response to supplementation. In this randomized clinical trial involving 600 euthyroid women with positive TPOAb undergoing IVF-ET, the miscarriage rate before 28 weeks’ gestation was 10.3% among women who received and 10.6% among those who did not receive LT4, a non-significant difference. Therefore, LT4 treatment did not appear to improve pregnancy outcomes in this patient population.

Before generalizing these results, however, it is important to have in mind some of the limitations of this well-conducted study. The incidence of miscarriage in clinically recognized pregnancies up to 20 gestational weeks is 8–20%; for women with thyroid autoimmunity, the miscarriage rate can be up to 30% (4). Taking this into consideration, ~10% miscarriage rate in this study reduces its power. As the authors outline, this lower miscarriage rate could be related to the fact that pregnant women with other comorbidities, such as positivity for anticardiolipin antibody, antinuclear antibody, or lupus anticoagulants, were excluded to eliminate other forms of autoimmunity as confounders. Given that this study included a selected population and patients with other conditions that increase the risk of miscarriage were excluded, it is still unknown whether these higher risk groups could potentially benefit from any unrecognized beneficial effect of LT4. Another consideration is the LT4 dosage. Current guidelines do not utilize clinical or biochemical variables to determine the LT4 starting dosage (9). This study took into account the participants’ baseline TSH level and weight to establish the administered LT4 dose. A recent study identified dose-dependent effects of TPOAb concentrations on thyroid function and the risk of premature delivery (10). It has been suggested that if thyroid autoimmunity is associated with adverse fecundity and fertility outcomes, this could be only in more severe cases. Thus, it would be important to assess whether there is a degree of clinically relevant thyroid autoimmunity that would indicate a treatment threshold. In the POSTAL study, a stratified analysis by TPOAb/TgAb level did not change the conclusions. Moreover, although differences in miscarriage rate appeared clinically relevant in multiple subgroup analyses (e.g., male infertility only), they did not reach statistical significance, which could be explained by the small sample size. Indeed, although a stratified analysis by TSH level >4.0 mIU/L did not show any benefit with regard to pregnancy loss between treated and untreated women, it is important to note that by study design there were only 17 women with TSH >4.0 mIU/L. This is particularly meaningful as a recent national US cohort found that pregnant women with TSH 4.1–10 mIU/L who received LT4 were less likely to experience a pregnancy loss compared to those who did not (11). In fact, the 2017 ATA guideline, taking into account all published data, strongly recommends LT4 treatment for TPOAb-positive women with a TSH greater than the pregnancy-specific reference range (or >4.0 mIU/L if unavailable) (9).

In conclusion, the POSTAL study showed that LT4 treatment did not benefit euthyroid women with positive TPOAb undergoing IVF-ET. This study adds important knowledge to the field, as LT4 therapy was started early in pregnancy allowing evaluation of early pregnancy outcomes. However, we still need more data to identify subgroups of women with normal thyroid function but positive thyroid antibodies who could potentially benefit from LT4 treatment. Clinicians should share the best available evidence regarding the risks/benefits of LT4 treatment with each pregnant woman enacting a patient-centered approach that takes into account their informed preferences when deciding if LT4 therapy should be started (12).

## Author Contributions

SM wrote the first draft of the manuscript. NSO and DOK wrote sections of the manuscript. All authors contributed to manuscript revision, read and approved the submitted version.

## Conflict of Interest Statement

The authors declare that the research was conducted in the absence of any commercial or financial relationships that could be construed as a potential conflict of interest.
